# Prognostic significance of spatial and density analysis of T lymphocytes in colorectal cancer

**DOI:** 10.1038/s41416-022-01822-6

**Published:** 2022-04-21

**Authors:** Hanna Elomaa, Maarit Ahtiainen, Sara A. Väyrynen, Shuji Ogino, Jonathan A. Nowak, Marjukka Friman, Olli Helminen, Erkki-Ville Wirta, Toni T. Seppälä, Jan Böhm, Markus J. Mäkinen, Jukka-Pekka Mecklin, Teijo Kuopio, Juha P. Väyrynen

**Affiliations:** 1 Department of Biological and Environmental Science, University of Jyväskylä, Jyväskylä, Finland; 2 Department of Education and Research, Central Finland Health Care District, Jyväskylä, Finland; 3 Department of Pathology, Central Finland Health Care District, Jyväskylä, Finland; 4 Department of Internal Medicine, Oulu University Hospital, Oulu, Finland; 5 Program in MPE Molecular Pathological Epidemiology, Department of Pathology, Brigham and Women’s Hospital and Harvard Medical School, Boston, MA USA; 6 Department of Epidemiology, Harvard T.H. Chan School of Public Health, Boston, MA USA; 7 Broad Institute of MIT and Harvard, Cambridge, MA USA; 8 Cancer Immunology and Cancer Epidemiology Programs, Dana-Farber Harvard Cancer Center, Boston, MA USA; 9 Surgery Research Unit, Cancer and Translational Medicine Research Unit, Medical Research Center Oulu, Oulu University Hospital, and University of Oulu, Oulu, Finland; 10 Department of Gastroenterology and Alimentary Tract Surgery, Tampere University Hospital, Tampere, Finland; 11 Department of Gastrointestinal Surgery, Helsinki University Hospital, Helsinki, Finland; 12 Applied Tumor Genomics Research Program, University of Helsinki, Helsinki, Finland; 13 Cancer and Translational Medicine Research Unit, Medical Research Center Oulu, Oulu University Hospital, and University of Oulu, Oulu, Finland; 14 Faculty of Sport and Health Sciences, University of Jyväskylä, Jyväskylä, Finland

**Keywords:** Tumour immunology, Cancer microenvironment, Colorectal cancer

## Abstract

**Background:**

Although high T cell density is a strong favourable prognostic factor in colorectal cancer, the significance of the spatial distribution of T cells is incompletely understood. We aimed to evaluate the prognostic significance of tumour cell-T cell co-localisation and T cell densities.

**Methods:**

We analysed CD3 and CD8 immunohistochemistry in a study cohort of 983 colorectal cancer patients and a validation cohort (*N* = 246). Individual immune and tumour cells were identified to calculate T cell densities (to derive T cell density score) and G-cross function values, estimating the likelihood of tumour cells being co-located with T cells within 20 µm radius (to derive T cell proximity score).

**Results:**

High T cell proximity score associated with longer cancer-specific survival in both the study cohort [adjusted HR for high (vs. low) 0.33, 95% CI 0.20–0.52, *P*_trend_ < 0.0001] and the validation cohort [adjusted HR for high (vs. low) 0.15, 95% CI 0.05–0.45, *P*_trend_ < 0.0001] and its prognostic value was independent of T cell density score.

**Conclusions:**

The spatial point pattern analysis of tumour cell-T cell co-localisation could provide detailed information on colorectal cancer prognosis, supporting the value of spatial measurement of T cell infiltrates as a novel, robust tumour-immune biomarker.

## Background

Colorectal cancer is the third most common cancer, covering around 10% of all new cancer cases worldwide [1]. The tumour microenvironment is composed of neoplastic tumour cells and non-neoplastic cells, such as host immune cells, interacting through cell–cell contacts and inflammatory mediators [2]. The assessment of colorectal cancer prognosis and treatment is mainly based on evaluating neoplastic tumour cells and tumour spread rather than analysing the host immune response [3]. The most widely used clinical staging system is the American Joint Committee on Cancer/International Union Against Cancer (AJCC/UICC) TNM classification, which includes the extent of the primary tumour (T), presence of lymph node metastasis (N) and spread to distant organs (M), while World Health Organization (WHO) histologic grading categorises tumours according to their differentiation [4]. However, these methods do not entirely capture the characteristics of colorectal tumours and their prognoses.

Immunoscore® is a T cell scoring system based on computer-assisted quantification of CD3^+^ and CD8^+^ cell densities in the tumour centre and the invasive margin [5, 6]. T cell density varies within a single tumour and between different tumours. The density is generally higher in the invasive margin than in the centre of the tumour. In mismatch repair (MMR)-deficient colorectal cancers, T cell densities are also usually higher than in MMR proficient tumours [7, 8]. High Immunoscore® has been associated with better prognosis and has been internationally validated as an independent prognostic parameter in a cohort of more than 2600 disease stage I–III colorectal cancer cases [6]. However, most studies evaluating immune cell infiltrates in colorectal cancer have been limited to density-based analyses [9] and the significance of the co-localisation between tumour cells and T cells is not well-established.

In this study, we used immunohistochemistry and digital image analysis to identify CD3^+^ and CD8^+^ cells and tumour cells in 1229 colorectal cancer samples, including a study cohort of 983 patients and an independent validation cohort of 246 cases. We present the T cell proximity score as a novel prognostic parameter based on the evaluation of co-localisation of tumour cells with T cells. Our primary aim was to evaluate the prognostic value of the T cell proximity score and compare it to that of the T cell density score (based on the principles of Immunoscore®). We hypothesised that a high T cell proximity score (high likelihood of tumour cells being co-located with T cells) might be associated with favourable outcome. As secondary aims, we investigated the associations of T cell proximity score with tumour and patient characteristics and the prognostic significance of spatial T cell proximity measurements separately in the tumour centre and the invasive margin, in MMR proficient and MMR deficient tumour subgroups as well as in low and high disease stage tumours.

## Methods

### Patients

We identified 1343 patients who underwent resection for colorectal cancer at Central Finland Central Hospital between January 1, 2000 and December 31, 2015 and had adequate tumour samples available. The samples were retrospectively collected from the pathology registry of Central Finland Central Hospital, which covers all colorectal cancers diagnosed in Central Finland (the population of the area-averaged around 270,000 during the study period) [10]. Associated clinical data were collected from clinical patient records by study physicians. We excluded patients who died within 30 days after surgery (*N* = 40) or received any preoperative oncological treatments (radiotherapy, chemotherapy or chemoradiotherapy) (*N* = 243) due to their potential influences on tumour characteristics [11]. The final cohort with adequate samples in tissue microarrays and successful quantification of CD3^+^ and CD8^+^ cells included 983 patients. The median follow-up time for censored was 9.3 years (IQR 6.8–13.3 years). The main clinicopathologic features of the cases are shown in Table 1. Histological tumour parameters were re-evaluated by the study pathologist (J.P.V.), including tumour differentiation and lymphovascular invasion, using hematoxylin and eosin (H&E) stained whole slides. All the histological analyses were performed blinded to the clinical data.

**Table 1:** Demographic and clinicopathologic characteristics of colorectal cancer cases according to T cell proximity score.

		T cell proximity score			
Characteristic	Total *N*	Low	Intermediate	High	*P*
All cases	983 (100%)	194 (20%)	545 (55%)	244 (25%)	
Sex					0.65
Female	481 (49%)	96 (49%)	260 (48%)	125 (51%)	
Male	502 (51%)	98 (51%)	285 (52%)	119 (49%)	
Age (years)					0.17
<65	265 (27%)	57 (29%)	142 (26%)	66 (27%)	
65–75	348 (35%)	58 (30%)	211 (39%)	79 (32%)	
>75	370 (38%)	79 (41%)	192 (35%)	99 (41%)	
Year of operation					0.26
2000–2005	299 (30%)	60 (31%)	153 (28%)	86 (35%)	
2006–2010	315 (32%)	65 (34%)	183 (34%)	67 (28%)	
2011–2015	369 38%)	69 (36%)	209 (38%)	91 (37%)	
Tumour location					0.0003
Proximal colon	478 (49%)	82 (42%)	249 (46%)	147 (60%)	
Distal colon	359 (40%)	83 (43%)	214 (39%)	62 (25%)	
Rectum	146 (15%)	29 (15%)	82 (15%)	35 (14 %)	
AJCC disease stage					<0.0001
I	162 (16%)	19 (10%)	84 (15%)	59 (24%)	
II	371 (38%)	62 (32%)	193 (35%)	116 (48%)	
III	322 (33%)	80 (41%)	186 (34%)	56 (23%)	
IV	128 (13%)	33 (17%)	82 (15%)	13 (5.3%)	
Tumour grade					0.061
Low-grade (well to moderately differentiated)	813 (83%)	166 (86%)	457 (84%)	190 (78%)	
High-grade (poorly differentiated)	170 (17%)	28 (14%)	88 (16%)	54 (22%)	
Lymphovascular invasion					<0.0001
No	772 (79%)	132 (68%)	423 (78%)	217 (89%)	
Yes	211 (21%)	62 (32%)	122 (22%)	17 (11%)	
MMR status					<0.0001
MMR proficient	833 (85%)	184 (95%)	485 (89%)	164 (67%)	
MMR deficient	150 (15%)	10 (5.2%)	60 (11%)	80 (33%)	
*BRAF* status					<0.0001
Wild-type	824 (84%)	177 (91%)	469 (86%)	178 (73%)	
Mutant	159 (16%)	17 (8.8%)	76 (14 %)	66 (27%)	

*AJCC* American Joint Committee on Cancer, *MMR* mismatch repair.

### Tissue microarrays

For tissue microarray construction, we selected one representative formalin-fixed paraffin-embedded tumour sample with the deepest cancer invasion for each patient. The arrays were constructed using a TMA Master II tissue microarrayer (3DHistech Ltd., Budapest, Hungary), and they included two 1 mm-diameter cores from representative areas of the tumour centre and the invasive margin (total: four cores). The core sites were annotated to best represent overall tumour morphology while avoiding necrosis. The invasive margin cores were targeted to span 500 µm into the healthy tissue and 500 µm into the tumour. In total, the cohort included 25 tissue microarray blocks, each containing two tonsil cores as staining controls. Tissue microarray blocks were tempered overnight at 60 °C and cut at 3.5 µm thickness.

### Immunohistochemistry

The samples were screened for DNA mismatch repair (MMR) deficiency with MLH1, MSH2, MSH6 and PMS2 immunohistochemistry and for *BRAF* V600E mutation status with immunohistochemistry [12]. Immunohistochemistry for T cells and MMR genes were performed by BOND-III automated IHC stainer (Leica Biosystems, Buffalo Grove, IL, USA) with monoclonal antibodies and protocols shown in Table S1. All antibodies were in clinical use in the pathology laboratory of Central Finland Central Hospital, and appropriate staining was also confirmed by examining positive and negative controls. After bake, dewax and peroxide block, slides were processed with heat-induced antigen retrieval with EDTA-based buffer, pH 9.0 (BOND Epitope retrieval solution 2, Leica Biosystems, AR9640) at 100 °C. Antigen retrieval time was 30 min for MLH1, MSH2, MSH6 and PMS2 and 20 min for CD3 and CD8. Primary antibodies were incubated for 30 min. Visualisation was performed according to the manufacturer’s instructions using a BOND Polymer Refine Detection kit (Leica Biosystems, DS9800) with a horseradish peroxidase-conjugated secondary antibody (<25 µg/ml), 3.3-diaminobenzidine chromogen and hematoxylin (0.1%) counterstain. Stained slides were coverslipped with a Tissue-Tek Glas Automated Glass Coverslipper (Sakura) and digitalised with a NanoZoomer-XR (Hamamatsu Photonics, Hamamatsu City, Japan) slide scanner with a ×20 objective.

Immunohistochemistry to evaluate *BRAF* V600E mutation status was conducted using a BenchMark XT immunostainer (Ventana Medical Systems, Tucson, AZ) and a BRAF V600E mutation-specific mouse monoclonal antibody (clone: VE1, Spring Bioscience, Pleasonton, CA, US, dilution: 1:400). The amplification was done with OptiView Amplification (Ventana [13]).

### Image analysis

T cell analyses were conducted with supervised machine learning approaches built-in QuPath (version 0.2.3), an open-source bioimage analysis software [14], using previously validated algorithms [15]. The software was trained to recognise tissue and cell types by manually annotating representative areas/cells. The identification of tissue from the background was done with the random forests *pixel classifier*. Cells were detected and classified into tumour cells, T cells, and other cells using the random forests *object classifier*. T cells were recognised by CD3 or CD8 expression and tumour cells were identified through their morphology. The remaining cells were classified as other. The workflow for image analysis is shown in Fig. S1. We confirmed the validity of the automated cell classifier by reviewing the classification result images. We further quantified the accuracy of the classifier by manually annotating each cell in 50 tumour regions (25 stained for CD3 and 25 stained for CD8; size 200 × 200 µm) and comparing the cell densities observed in these regions with those obtained with the automated classifier using the Spearman’s rank correlation test.

All tissue microarray cores were reviewed, and those with folding or detaching during processing, minimal amount or absence of tumour, or high amount of necrosis were excluded from the analyses. For the final analyses, we included only cases that at least one representative successfully analysed the tumour core from the tumour centre and the invasive margin for both CD3 and CD8. For each tissue microarray core, we calculated CD3^+^ and CD8^+^ cell densities by dividing the cell counts by the tumour core area in mm^2^. For tumours with multiple successfully analysed tumour centre cores or invasive margin cores, we calculated mean cell densities. As a result, each tumour had one density value for (1) CD3^+^ cells in the tumour centre, (2) CD3^+^ cells in the invasive margin, (3) CD8^+^ cells in the tumour centre and (4) CD8^+^ cells in the invasive margin.

**Fig. 1:**
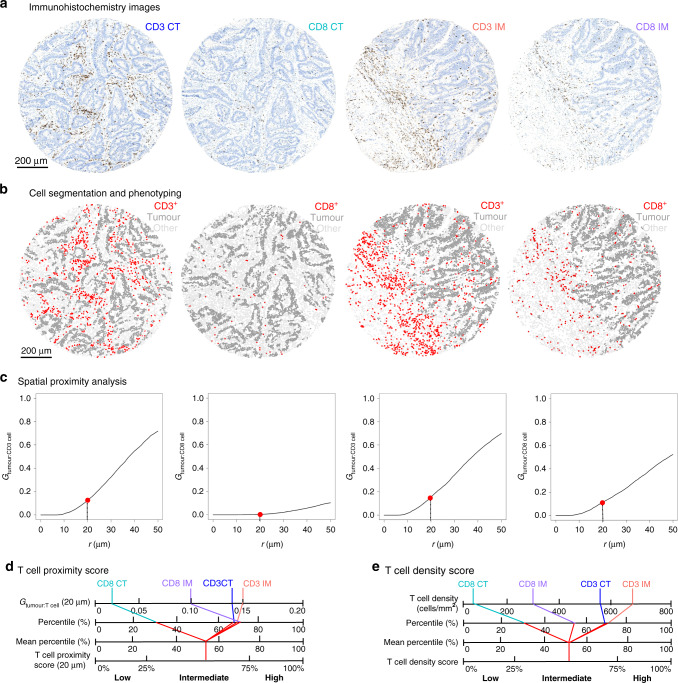
T cell proximity and density score analyses in colorectal cancer. The figure shows analysis steps for one example tumour core from the tumour centre (CT) and the invasive margin (IM). Tumour cores stained with CD3 and CD8 (**a**) and corresponding phenotyping maps for T cells, tumour cells and other cells (**b**). G-cross (G_tumour:T cell_) function curves, representing the likelihood of any tumour cell in the sample being co-located with at least one CD3^+^/CD8^+^ T cell within a radius r (**c**). Calculation charts for T cell proximity score (**d**) and for T cell density score (**e**). Respective example images for two remaining cores of the same sample are shown in Fig. S2.

To calculate the T cell density score, we followed the main principles of the Immunoscore assay [6]. The densities of CD3^+^ cells in the tumour centre, CD3^+^ cells in the invasive margin, CD8^+^ cells in the tumour centre and CD8^+^ cells in the invasive margin were converted to percentiles (0–100), which resulted in four separate percentile values for each tumour. T cell density score was determined by calculating the mean of the four percentiles and categorising it to low (0–25), intermediate (>25–70) or high (>70–100). The workflow for T cell density score analysis is shown in Fig. 1.

In this study, we introduced the T cell proximity score as a new prognostic parameter based on tumour cell-T cell co-localisation. We estimated the empirical G-cross [G_tumour:T cell_ (r)] function for each sample, evaluating the likelihood of any tumour cell in the sample having at least one T cell at a specific radius r. The function is formed by measuring the distances from each tumour cell centroid to the closest immune cell centroid. Thus, higher G-cross function values result from a higher percentage of tumour cells harbouring T cells in their proximity and indicate greater co-localisation of tumour cells with T cells. We chose to examine the function values at 20 µm radius to identify T cell populations likely capable of effective, direct, cell-to-cell interaction with tumour cells, consistent with previous reports [15–18]. We applied the Kaplan–Meier correction for edge effects. T cell proximity score was calculated using a similar approach as in T cell density score. We calculated G-cross [G_tumour:T cell_ (20 µm)] function values for CD3^+^ and CD8^+^ cells in the tumour centre and in the invasive margin and converted these four values into percentiles. To determine the T cell proximity score for each tumour, we calculated the mean of the four percentiles and categorised it into low (0–25), intermediate (>25–70) or high (>70–100). In sensitivity analysis, we tested the T cell proximity score at G-cross function radii of 10, 30, 40, 50, 100 and 500 µm. The workflow for the proximity score analysis for two example cores of the same tumour is shown in Fig. 1. Figure S2 represents the respective analysis for the two remaining cores of the example tumour.

### Validation cohort

For validation, we retrospectively analysed an independent, previously described colorectal cancer cohort operated at Oulu University Hospital from 2006 to 2014 [19]. Patients with preoperative treatment and unsuccessful CD3^+^ or CD8^+^ cell analysis were excluded, and the final data included 246 patients. The median follow-up time for censored cases was 6.2 years (IQR 5.0–7.6 years). Analyses were conducted for one to four 3 mm-diameter TMA cores per patient [19]. Antibodies and staining protocols are shown in Table S2. The densities and G-cross function values were compared with those obtained in the study cohort to convert them into percentiles.

### Statistical analyses

Statistical analyses were performed using RStudio (version 1.3.1093) and R statistical programming (version 4.0.5, R Core Team) with packages *gmodels* (2.18.1), *spatstat* (2.1-0), *survival* (3.2-7), *survminer* (0.4.9) and *tidyverse* (1.3.0).

Categorical data were analysed by cross-tabulation of T cell scores and other variables and using Chi-square test to evaluate the statistical significance. Kaplan–Meier method was used for visualising the cumulative survival probabilities, and the comparison between categories was done with the Log-rank test. As our primary analyses, we utilised univariable and multivariable Cox proportion hazard regression to estimate mortality hazard ratio (HR) point estimates and their 95% confidence intervals (CIs). Cancer-specific survival was evaluated as the primary endpoint, and it was defined as the time from surgery to cancer death. Overall survival was evaluated as the secondary endpoint, and it was defined as the time between colorectal cancer surgery and death. We limited the follow-up to 10 years, considering that most colorectal cancer deaths occur within that period. Schoenfeld residual plots supported the proportionality of hazards during most of the follow-up period up to 10 years. Multivariable models included the following pre-determined indicator variables (with the reference category listed first): sex (male, female), age (<65, 65–75, >75), year of operation (2000–2005, 2006–2010, 2011–2015), tumour location (proximal colon, distal colon, rectum), disease stage (I–II, III, IV), tumour grade (well/moderately differentiated, poorly differentiated), lymphovascular invasion (negative, positive), MMR status (proficient, deficient), *BRAF* status (wild-type, mutant). Cases with missing data (validation cohort only) were included in the majority category of a given categorical covariate to limit the degrees of freedom. The following covariates had missing values in the validation cohort: disease stage (0.4% missing), differentiation (0.4% missing), lymphovascular invasion (1.2% missing), MMR status (0.4% missing). Excluding those missing cases in each covariate did not substantially alter results. We used a stringent alpha level of 0.005 according to the recommendation of an expert panel [20].

## Results

### Patient characteristics

We analysed T cell infiltrates in tumour samples of 983 colorectal cancer patients. Of the patients, 481 (49%) were women and the median age at the time of surgery was 72 years (range 36–100 years). The most prevalent site for the primary tumour was the proximal colon (cecum to transverse colon) with 478 (49%) cases. MMR deficiency was detected in 150 (15%) tumours (Table 1).

### Cell analysis of tissue microarray cores

We successfully analysed a total of 2,351,513 CD3^+^ cells from 3632 tissue microarray cores and 1,105,424 CD8^+^ cells from 3608 tissue microarray cores. The average number of analysed cores was 3.7 per patient for both CD3 and CD8. Core-to-core correlations for G-cross (G_tumour:T cell_) function values at 20 µm radius were 0.69 for CD3^+^ cells in the tumour centre 0.70 for CD3^+^ cells in the invasive margin, 0.69 for CD8^+^ cells in the tumour centre and 0.70 for CD8^+^ cells for the invasive margin (Fig. S3), being slightly higher than the respective core-to-core correlations for T cell densities (Fig. S4).

We tested the accuracy of machine-learning-based image analysis by manually annotating all cells (T cells, tumour cells and other cells) in 50 tumour images and then applying the optimised automated classifier to these images. The total number of detected cells was 11,312 in manual counting and 12,097 in automated cell counting. The Spearman’s rank correlations coefficient between automated and manual cell densities was 0.94 for T cells, 0.87 for tumour cells and 0.86 for other cells, indicating that the classifier had reached good accuracy (Fig. S5).

### T cell proximity and density score

T cell proximity score was calculated based on tumour cell-T cell co-localisation measurements, using G_tumour:T cell_ function values at 20 µm (Fig. 1). Examples of tissue microarray cores with distinct T cell infiltration patterns and corresponding G-cross function curves are shown in Fig. S6. Of the patients, 545 (55%) cases were classified as intermediate for the proximity score, whereas low covered 20% and high covered 25% of the cases. High T cell proximity score was strongly associated with proximal tumour location (*P* = 0.0003), low disease stage, absence of lymphovascular invasion, MMR deficiency and *BRAF* mutation (all *P* < 0.0001; Table 1).

T cell density score was calculated based on the mean CD3^+^ and CD8^+^ cell densities in the tumour centre and the invasive margin according to the principles of Immunoscore® (Fig. 1). Like the proximity score, high density score was associated with proximal tumour location (*P* = 0.003), low disease stage (*P* = 0.0002), absence of lymphovascular invasion (*P* = 0.0004), MMR deficiency (*P* < 0.0001) and *BRAF* mutation (*P* = 0.001; Table S3).

### Survival analyses

In total, there were 574 (58%) deaths including 278 (28%) colorectal cancer deaths. The 5-year and 10-year cancer-specific survival rates were 74% and 69% and overall survival rates were 61% and 46%, respectively.

**Fig. 2:**
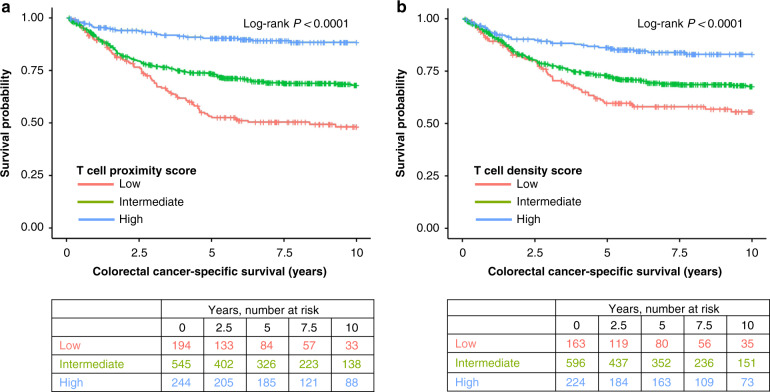
Kaplan-Meier estimates of colorectal cancer-specific survival. Kaplan–Meier cancer-specific survival curves for T cell proximity score (**a**) and T cell density score (**b**). Log-rank test was used to estimate the statistical significance.

Our primary aim was to evaluate the prognostic significance of the T cell proximity score and compare it to that of the T cell density score. High proximity and density scores predicted improved outcomes compared to low scores. The 10-year cancer-specific survival for patients with high and low proximity scores were 88% and 48%, respectively (Fig. 2, Table 2). High T cell proximity and density scores were associated with better cancer-specific and overall survival both in univariable and multivariable analyses. In cancer-specific survival analysis, the multivariable HR for high (vs. low) T cell proximity score was 0.33 (95% CI 0.20–0.52, *P*_trend_ < 0.0001) and for high (vs. low) density score 0.47 (95% CI 0.31–0.73, *P*_trend_ = 0.0007) (Table 2, Table S4).

**Table 2:** Univariable and multivariable Cox regression models for cancer-specific survival and overall survival according to T cell proximity score and T cell density score.

		Colorectal cancer-specific survival			Overall survival		
	No. of cases	No. of events	Univariable HR (95% CI)	Multivariable HR (95% CI)	No. of events	Univariable HR (95% CI)	Multivariable HR (95% CI)
T cell proximity score							
Low	194	88	1 (referent)	1 (referent)	127	1 (referent)	1 (referent)
Intermediate	545	157	0.57 (0.44–0.75)	0.72 (0.55–0.94	269	0.66 (0.53–0.81)	0.74 (0.59–0.91)
High	244	25	0.18 (0.12–0.29)	0.33 (0.20–0.52)	98	0.47 (0.36–0.61)	0.57 (0.43–0.76)
* P*_trend_			<0.0001	<0.0001		<0.0001	0.0001
T cell density score							
Low	163	64	1 (referent)	1 (referent)	98	1 (referent)	1 (referent)
Intermediate	596	172	0.69 (0.52–0.93)	0.74 (0.55–0.99)	302	0.78 (0.62–0.99)	0.78 (0.62–0.99)
High	224	34	0.34 (0.22–0.51)	0.47 (0.31–0.73)	94	0.59 (0.44–0.78)	0.62 (0.46–0.84)
* P*_trend_			<0.0001	0.0007		0.0002	0.002

Multivariable Cox proportional hazards regression models were adjusted for sex, age (<65, 65–75, >75), year of operation (2000–2005, 2006–2010, 2011–2015), tumour location (proximal colon, distal colon, rectum), disease stage (I–II, III, IV), tumour grade (well/moderately differentiated, poorly differentiated), lymphovascular invasion (negative, positive), MMR status (proficient, deficient), *BRAF* status (wild-type, mutant).

*P*_trend_ values were calculated by using the three ordinal categories of T cell proximity score and T cell density score as continuous variables in univariable and multivariable Cox proportional hazard regression models.

*CI* confidence interval, *HR* hazard ratio.

To directly compare the prognostic value of T cell proximity and density scores for cancer-specific survival, we included these variables in the same multivariable Cox regression model for reciprocal adjustment (Table 3). This analysis indicated that the prognostic significance of the proximity score (*P*_trend_ = 0.001) was independent of the density score (*P*_trend_ = 0.75).

**Table 3:** Comparison of prognostic power of T cell proximity score and T cell density score using Cox regression models for cancer-specific survival.

	No. of cases	No. of events	Model 1 (univariable) HR (95% CI)	Model 2 (multivariable) HR (95% CI)	Model 3 (multivariable) HR (95% CI)
T cell proximity score					
Low	194	88	1 (referent)	1 (referent)	1 (referent)
Intermediate	545	157	0.57 (0.44–0.75)	0.52 (0.38–0.73)	0.75 (0.54–1.04)
High	244	25	0.18 (0.12–0.29)	0.15 (0.08–0.27)	0.32 (0.17–0.60)
* P*_trend_			<0.0001	<0.0001	0.001
T cell density score					
Low	163	64	1 (referent)	1 (referent)	1 (referent)
Intermediate	596	172	0.69 (0.52–0.93)	1.15 (0.81–1.65)	0.91 (0.64–1.30)
High	224	34	0.34 (0.22–0.51)	1.33 (0.75–2.34)	1.01 (0.56–1.81)
* P*_trend_			<0.0001	0.35	0.75

Model 2: Cox proportional hazards regression model including T cell proximity score and T cell density score.

Model 3: Cox proportional hazards regression model based on Model 2 that was additionally adjusted for sex, age (<65, 65–75, >75), year of operation (2000–2005, 2006–2010, 2011–2015), tumour location (proximal colon, distal colon, rectum), disease stage (I–II, III, IV), tumour grade (well/moderately differentiated, poorly differentiated), lymphovascular invasion (negative, positive), MMR status (proficient, deficient), *BRAF* status (wild-type, mutant).

*P*_trend_ values were calculated by using the three ordinal categories of T cell proximity score and T cell density score as continuous variables in univariable and multivariable Cox proportional hazard regression models.

*CI* confidence interval, *HR* hazard ratio.

Of the proximity score high cases (*N* = 244), 179 (73%) cases were high and 65 (27%) were intermediate for the density score. Of the proximity score low cases (*N* = 196), 121 (62%) were low and 75 (38%) were intermediate for the density score. We categorised the tumours into four subgroups to evaluate the combined prognostic effect of the proximity and density scores (Fig. S7, Table S5). This analysis further indicated that a high T cell proximity score was associated with better survival regardless of the density score.

In secondary analyses, we evaluated the survival associations of the four components (CD3^+^ and CD8^+^ cells in the tumour centre and the invasive margin) of T cell proximity [(G_tumour:T cell_) at a 20 µm radius] and density scores as ordinal quartile categories (Table 4, Fig. S8). In this analysis, higher values in all four components of both T cell proximity and T cell density score, except CD8^+^ cell density in the tumour centre, were statistically significantly associated with longer cancer-specific survival (all *P*_trend_ < 0.005). The HR point estimates suggested stronger survival associations for the measurements based on the invasive margin, as compared to the tumour centre, and for the G-cross proximity estimates, as compared to the densities.

**Table 4:** Univariable and multivariable Cox regression models for cancer-specific and overall survival according to G-cross (G_tumour:T cell_) proximity function values at 20 µm radius and T cell densities.

		Colorectal cancer-specific survival				Overall survival		
	No. of cases	No. of events	Univariable HR (95% CI)	Multivariable HR (95% CI)		No. of events	Univariable HR (95% CI)	Multivariable HR (95% CI)
*CD3*^*+*^ *cell proximity*								
Tumour center								
Q1	246	100	1 (referent)		1 (referent)	152	1 (referent)	1 (referent)
Q2	246	82	0.75 (0.56–1.00)		0.88 (0.65–1.18)	135	0.81 (0.64–1.02)	0.86 (0.68–1.08)
Q3	246	55	0.48 (0.34–0.67)		0.65 (0.46–0.91)	114	0.64 (0.50–0.81)	0.75 (0.58–0.96)
Q4	245	33	0.26 (0.18–0.39)		0.45 (0.30–0.68)	93	0.47 (0.36–0.61)	0.57 (0.43–0.75)
* P*_trend_			<0.0001		<0.0001		<0.0001	<0.0001
Invasive margin								
Q1	246	106	1 (referent)		1 (referent)	158	1 (referent)	1 (referent)
Q2	246	91	0.84 (0.63–1.11)		0.93 (0.70–1.24)	137	0.84 (0.67–1.06)	0.88 (0.70–1.11)
Q3	246	45	0.35 (0.25–0.50)		0.61 (0.42–0.88)	94	0.48 (0.37–0.62)	0.66 (0.50–0.86)
Q4	245	28	0.22 (0.15–0.34)		0.35 (0.22–0.55)	105	0.53 (0.42–0.68)	0.62 (0.47–0.81)
* P*_trend_			<0.0001		<0.0001		<0.0001	<0.0001
*CD8*^*+*^ *cell proximity*								
Tumour center								
Q1	246	95	1 (referent)		1 (referent)	146	1 (referent)	1 (referent)
Q2	246	82	0.85 (0.63–1.15)		1.08 (0.80–1.46)	135	0.91 (0.72–1.14)	1.04 (0.82-1.32)
Q3	246	57	0.54 (0.39–0.75)		0.77 (0.55–1.08)	110	0.65 (0.51–0.84	0.81 (0.63–1.04)
Q4	245	36	0.33 (0.22–0.48)		0.59 (0.39–0.89)	103	0.58 (0.45–0.74)	0.75 (0.57–0.98)
* P*_trend_			<0.0001		0.006		<0.0001	0.012
Invasive margin								
Q1	246	110	1 (referent)		1 (referent)	151	1 (referent)	1 (referent)
Q2	246	80	0.70 (0.53–0.94)		0.70 (0.52–0.94)	135	0.85 (0.67–1.07)	0.90 (0.71–1.14)
Q3	246	49	0.38 (0.27–0.53)		0.57 (0.40–0.82)	99	0.54 (0.42–0.70)	0.71 (0.55–0.93)
Q4	245	31	0.24 (0.16–0.35)		0.38 (0.25–0.58)	109	0.59 (0.46–0.75)	0.70 (0.53–0.91)
* P*_trend_			<0.0001		<0.0001		<0.0001	0.003
*CD3*^*+*^ *cell density*								
Tumour center								
Q1	246	92	1 (referent)		1 (referent)	151	1 (referent)	1 (referent)
Q2	246	85	0.88 (0.66–1.19)		1.17 (0.86–1.59)	135	0.85 (0.67–1.07)	1.14 (0.89–1.45)
Q3	246	54	0.50 (0.36–0.70)		0.71 (0.50–1.00)	105	0.58 (0.45–0.75)	0.79 (0.61–1.02)
Q4	245	39	0.31 (0.25–0.52)		0.52 (0.35–0.76)	103	0.56 (0.43–0.71)	0.67 (0.52–0.87)
* P*_trend_			<0.0001		0.0001		<0.0001	0.0003
Invasive margin								
Q1	246	98	1 (referent)		1 (referent)	152	1 (referent)	1 (referent)
Q2	246	81	0.78 (0.58–1.05)		0.92 (0.68–1.24)	127	0.78 (0.61–0.98)	0.89 (0.70–1.13)
Q3	246	55	0.50 (0.36–0.69)		0.66 (0.47–0.93)	114	0.65 (0.51–0.83)	0.75 (0.58–0.96)
Q4	245	36	0.31 (0.21–0.45)		0.48 (0.32–0.71)	101	0.54 (0.42–0.69)	0.61 (0.47–0.80)
* P*_trend_			<0.0001		<0.0001		<0.0001	0.0002
*CD8*^*+*^ *cell density*								
Tumour center								
Q1	246	90	1 (referent)		1 (referent)	145	1 (referent)	1 (referent)
Q2	246	78	0.84 (0.62–1.13)		0.97 (0.71–1.31)	128	0.85 (0.67–1.08)	0.95 (0.74–1.20)
Q3	246	57	0.59 (0.42–0.82)		0.74 (0.52–1.04)	117	0.73 (0.57–0.93)	0.83 (0.65–1.06)
Q4	245	45	0.44 (0.31–0.63)		0.65 (0.45–0.95)	104	0.61 (0.48–0.79)	0.73 (0.56–0.95)
* P*_trend_			<0.0001		0.010		<0.0001	0.011
Invasive margin								
Q1	246	105	1 (referent)		1 (referent)	155	1 (referent)	1 (referent)
Q2	246	62	0.51 (0.37–0.69)		0.73 (0.53–1.02)	112	0.60 (0.47–0.76)	0.76 (0.59–0.97)
Q3	246	61	0.51 (0.37–0.70)		0.65 (0.47–0.90)	114	0.64 (0.50–0.81)	0.72 (0.56–0.93)
Q4	245	42	0.34 (0.24–0.49)		0.53 (0.37–0.78)	113	0.61 (0.48–0.78)	0.72 (0.56–0.94)
* P*_trend_			<0.0001		0.0004		0.0003	0.013

Analyses were done separately for CD3^+^ and CD8^+^ cells in tumour center and in invasive margin by using ordinal quartile categories (Q1–Q4, from low to high).

Multivariable Cox proportional hazards regression models were adjusted for sex, age (<65, 65–75, >75), year of operation (2000–2005, 2006–2010, 2011–2015), tumour location (proximal colon, distal colon, rectum), disease stage (I–II, III, IV), tumour grade (well/moderately differentiated, poorly differentiated), lymphovascular invasion (negative, positive), MMR status (proficient, deficient), *BRAF* status (wild-type, mutant).

*P*_trend_ values were calculated by using the four ordinal categories of G-cross (G_tumour:T cell_) proximity function values at 20 µm radius and T cell densities as continuous variables in univariable and multivariable Cox proportional hazard regression models.

*CI* confidence interval, *HR* hazard ratio.

For sensitivity analysis, modified T cell proximity scores were derived from G_tumour:T cell_ function values at different radii (10–50, 100 and 500 µm) (Table S6). Univariable and multivariable Cox regression models for cancer-specific survival indicated strong prognostic associations for the proximity scores at 10–50 µm and 100 µm radii (all *P*_trend_ < 0.0001), but not at 500 µm radius (*P*_trend_ = 0.22). These results support the significance of tumour cell-T cell co-localisation within radii of 10–100 µm.

To further evaluate factors potentially influencing the prognostic significance of the T cell proximity score, we investigated the prognostic effect of the proximity score in MMR proficient and deficient tumour subgroups, as well as in different disease stages. The association between a higher T cell proximity score and longer cancer-specific survival did not significantly differ by MMR status (*P*_interaction_ = 0.69) (Table S7), while a higher T cell proximity score was associated with longer cancer-specific survival in stages I–III but not in stage IV (*P*_interaction_ < 0.0001) (Fig. S9, Tables S8, S9).

### Validation cohort

We analysed an independent validation cohort of 246 patients. The clinicopathologic features for the validation cohort (*N* = 246) are shown in Table S10. High T cell proximity score was associated with low disease stage (*P* < 0.0001), absence of lymphovascular invasion (*P* = 0.0002), MMR deficiency (*P* < 0.0001) and *BRAF* mutation (*P* = 0.0002).

In total, there were 80 (33%) deaths including 58 (24%) colorectal cancer deaths. In this cohort, T cell proximity score was associated with longer cancer-specific survival [multivariable HR for high (vs. low) 0.15, 95% CI 0.05–0.45, *P*_trend_ < 0.0001; Table S11]. As in the main cohort, the prognostic association of the proximity score was independent of the density score (Table S12).

## Discussion

We investigated the spatial distribution and density of CD3^+^ and CD8^+^ cells in a large, population-based cohort of 983 colorectal cancer patients and an independent validation cohort of 246 colorectal cancer cases. T cell density is a well-established favourable prognostic parameter in colorectal cancer [9], but relatively few studies [15–18, 21–24] have investigated the role of the spatial organisation of immune cell infiltrates in cancer. G-cross function has been previously used for analyzing the spatial interactions of T cells and tumour cells in primary tumours [15, 17] and in liver metastases [22] of colorectal cancer, in non-small cell lung cancer [16] and in pancreatic cancer [18]. In this study, we utilised CD3 and CD8 immunohistochemistry and quantified G-cross (G_tumour:T cell_) function values at 20 μm radius at the invasive margin and tumour centre and established T cell proximity score as a new, reproducible system for analyzing the co-localisation of tumour cells with T cells. Our main finding was that a high T cell proximity score was associated with favourable outcomes independent of potential confounding factors such as disease stage and MMR status, as well as T cell density score. We envision that the method would be applicable to the analysis of a variety of other solid tumours and could be used as a quantitative tumour-immune biomarker, evaluating not only the density but also the spatial patterns of T cell infiltrates in the tumour.

Our findings were consistent with a recent study in colorectal cancer, where the co-localisation of tumour cells with CD3^+^ T cells within 20 μm radius was a stronger prognostic factor than total T cell density in the tumour microenvironment [17]. Moreover, previous studies have demonstrated that strong engagement and mixing of CD8^+^ cytotoxic T cells with tumour cells in colorectal cancer liver metastases are associated with favourable outcomes [21, 22]. In our study of primary colorectal cancer, the prognostic value of the spatial measurements for CD3^+^ cells was as significant as those for CD8^+^ cells both in the tumour centre and in the invasive margin. The present study represents a comprehensive analysis of tumour cell-T cell co-localisation in two large colorectal cancer cohorts, with detailed clinicopathologic characterisation [17].

T cell proximity score, introduced in this study, specifically evaluates the co-localisation of tumour cells with T cells within 20 µm radius, ignoring T cells located further from tumour cells. In our analyses, the proximity score had a higher prognostic value than the density score. We hypothesise that this may be related to the proximity score focusing on T cells with the potential for direct cell–cell interactions with tumour cells, such as cytotoxicity [15, 16, 18, 24]. It is conceivable that distant T cells may have a reduced possibility for anti-tumoural activity compared with T cells in close tumour proximity. Moreover, a high stromal percentage predicts an unfavourable prognosis in colorectal cancer, while a low stromal percentage is associated with favourable outcomes [25, 26]. Considering that the majority of immune cells in the colorectal cancer microenvironment are located in tumour stromal rather than intraepithelial regions [15, 27, 28], the tumours with low stroma percentage might have low overall T cell densities as a result of low stromal content rather than a weak anti-tumour-immune response, while spatial point pattern analysis may still classify these tumours into higher T cell proximity score categories if T cells are located close to tumour cells. All four components of the proximity score (CD3^+^ and CD8^+^ cells in tumour centre and in invasive margin) had strong prognostic significance as separate variables. In addition, our sensitivity analyses showed that high G-cross (G_tumour:T cell_) function values were associated with better prognosis within a range of radii between 10–50 µm and 100 µm, but not at 500 µm. These findings further highlight the robustness of the analysis, not dependent on a single, specific radius or component, and supports the potential of T cell proximity score as a relevant prognostic factor in colorectal cancer.

We identified the cell types with a machine-learning-based cell classifier using the QuPath software. We confirmed the adequacy of the classifier by manually viewing all result images and tested the accuracy of our cell classifier by comparing the densities of manually annotated cells and automatedly classified cells, supporting high concordance for all three cell types (T cells, tumour cells, and other cells). The machine-learning-based cell analysis for immunohistochemically stained tumour tissue samples using QuPath has also been validated in previous studies with high accuracy [15, 29, 30].

Some limitations should be considered. First, we used tissue microarrays, which may not totally represent the immunological milieu in the whole tumour [31, 32]. However, we successfully analysed on average 3.7 tumour cores for each patient, which should demonstrate relatively good concordance with the whole tumour [31]. We also observed reasonably good core-to-core correlation for both G-cross and density measurements, suggesting that T cell infiltrates can be evaluated using our tissue microarrays with reasonable accuracy. Moreover, measurement errors related to tissue microarrays would likely have a nearly random distribution, driving our findings towards the null hypothesis. Tissue microarrays also enabled staining of all samples at the same time, so the staining quality was uniform between the specimens. Second, the information on cancer treatment was lacking. Nevertheless, treatments have likely been principally based on the disease stage and MMR status rather than immune infiltrate, and we adjusted the multivariable survival models for several factors, including disease stage and MMR status. Third, we excluded all patients with preoperative treatment from analyses, which led to the under-representation of rectal cancers in these cohorts. The prognostic significance of the T cell proximity score should be interpedently evaluated in rectal cancer patients who have received neoadjuvant treatments. Fourth, although T cells play a critical role in anti-tumoural immunity, this study lacks the prognostic information of other immune cells in the tumour microenvironment. Fifth, most patients were non-Hispanic White, and the prognostic significance of the T cell proximity score should be confirmed in different populations.

There were several strengths in the study. This study included a large, thoroughly analysed study cohort [12, 33–36], as well as an independent validation cohort, which can forward the generalisability of the findings. The histological parameters were evaluated uniformly in accordance with the latest guidelines and the tumours were screened for two key molecular prognostic parameters (MMR status and *BRAF* mutation status). The machine learning assessment of immune cell infiltrates enabled uniform analysis throughout cases and spatial point pattern analyses based on positions of single cells [15]. This facilitated more detailed analyses of tumour cell-immune cell co-localisation than possible using traditional methods.

In conclusion, this study showed that the T cell proximity score, derived from G-cross measurements of co-localisation of tumour cells with T cells, was strongly associated with the survival of colorectal cancer patients, representing a new, quantitative prognostic parameter for colorectal cancer. Our results highlight the importance of the spatial context in the analysis of immune cell infiltrates in cancer and could be utilised to develop improved tumour-immune biomarkers for precision medicine.

## Supplementary information

Supplementary material

Reproducibility checklist

The REMARK checklist

## Data Availability

The datasets generated and/or analysed during this study are not publicly available. The sharing of data will require approval from relevant ethics committees and/or biobanks. Further information including the procedures to obtain and access data of Finnish Biobanks are described at https://finbb.fi/en/fingenious-service.
