# Supplementary material for: Prognostic significance of spatial and density analysis of T lymphocytes in colorectal cancer
Source: Br J Cancer. 2022 Apr 21;127(3):514–23. doi: 10.1038/s41416-022-01822-6 (PMC9345858; doi:10.1038/s41416-022-01822-6)
Supplement: Supplementary file 1 — Supplementary material [file 41416_2022_1822_MOESM1_ESM.pdf]

Supplementary figures

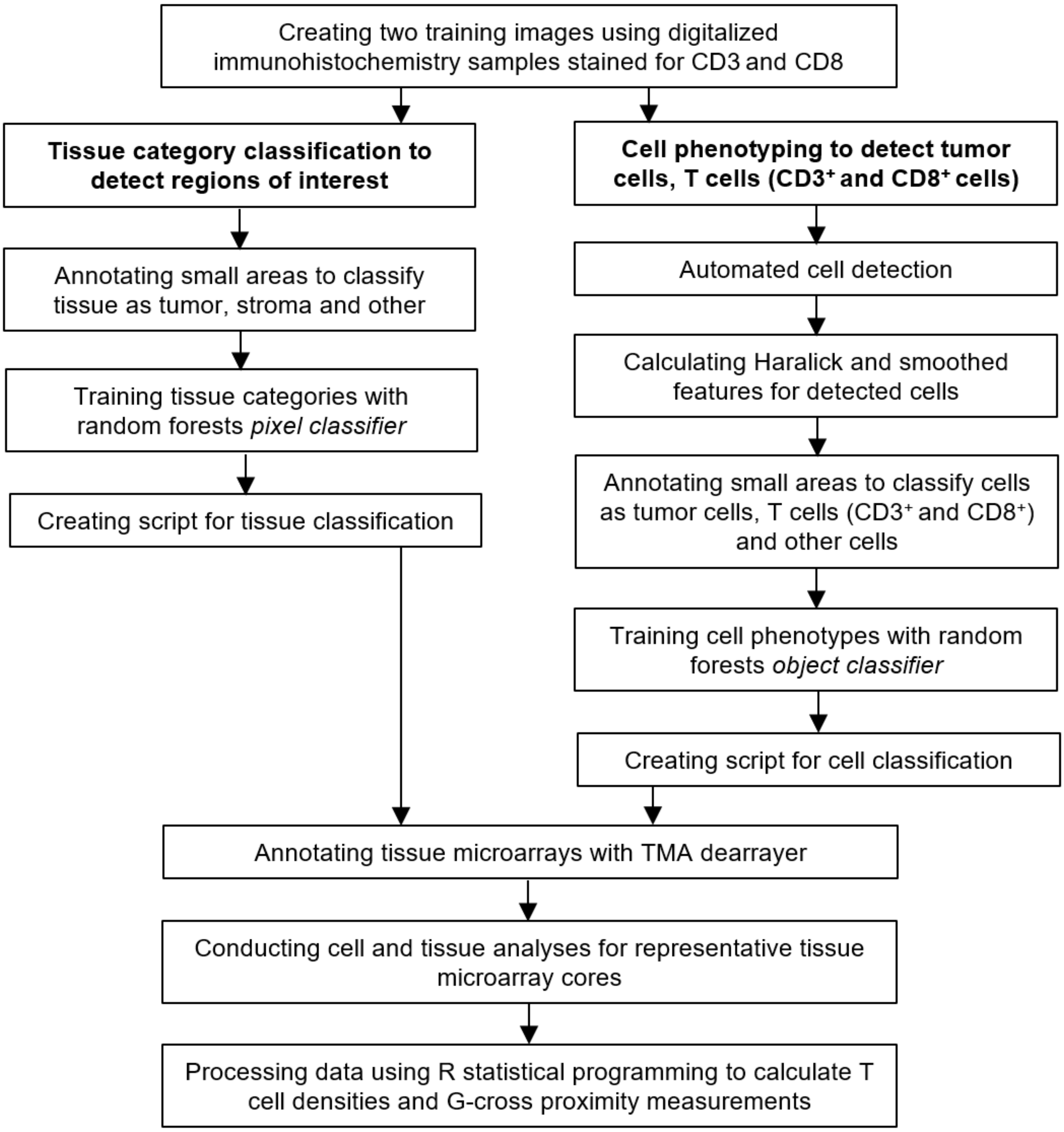

Figure S1 Image processing flowchart for $CD3^{+}$ and $CD8^{+}$ immunohistochemistry samples using machine learning algorithms of QuPath bioimage analysis software.

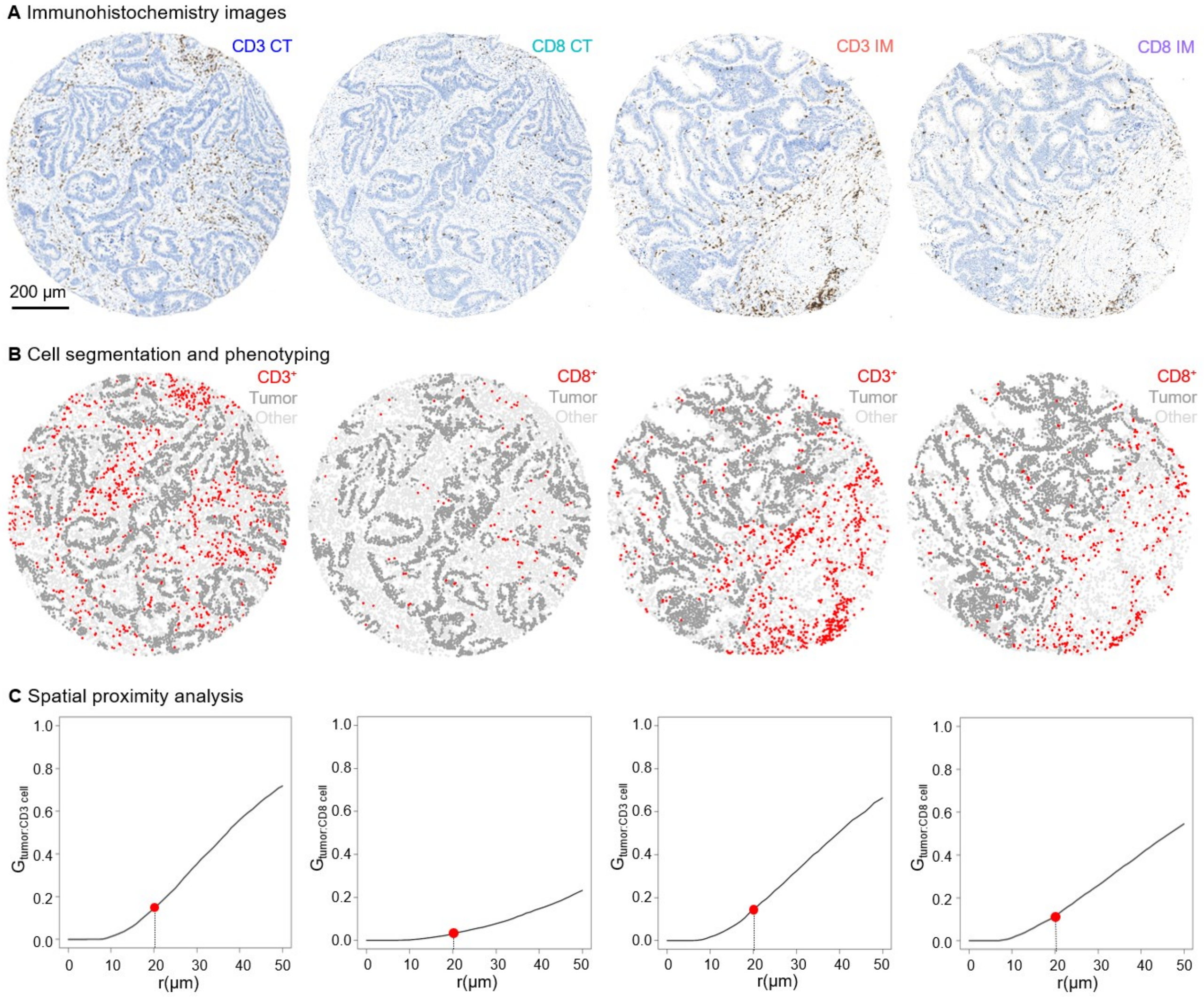

Figure S2 T cell proximity and density score analyses for two cores (one from tumor center (CT) and one from invasive margin (IM)) not represented in Figure 1. The panels show tumor cores stained with CD3 and CD8 (A), corresponding phenotyping maps for T cells, tumor cells and other cells (B), G-cross [$G_{tumor:T\ cell}$] function curves representing the likelihood of any tumor cell in the sample having at least one $CD3^+$/$CD8^+$ T cell within a radius r (C).

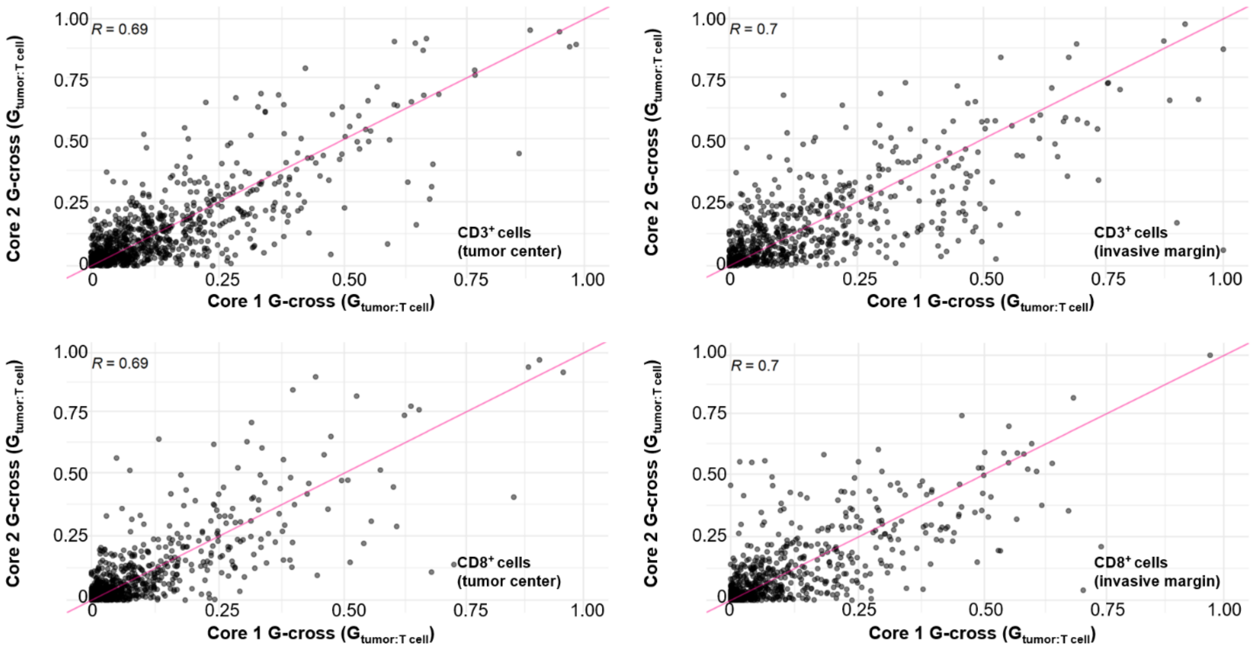

Figure S3 Core-to-core correlation of G-cross ($G_{tumor:T\ cell}$) at 20 μm radius in two randomly chosen cores of tumors with two or more cores measured using Spearman's rank correlation coefficients (*R*). Red line depicts perfect concordance (slope=1). G-cross correlations for $CD3^+$ and $CD8^+$ cells are presented separately in the tumor center and in the invasive margin. N=913 for CD3 in the tumor center, N=725 for CD3 in the invasive margin, N=877 for CD8 in the tumor center, N=738 for CD8 in the invasive margin.

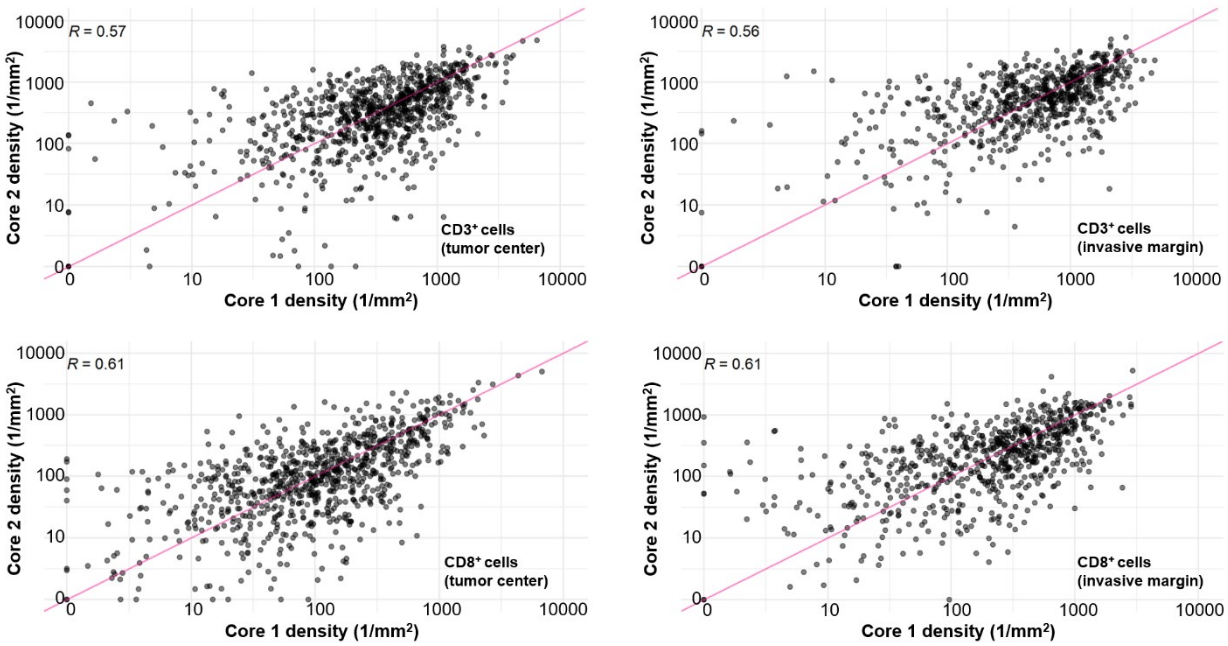

Figure S4 Core-to-core correlation of T cell densities in two randomly chosen cores of tumors with two or more cores measured using Spearman's rank correlation coefficients (*R*). Red line depicts perfect concordance (slope=1). $CD3^+$ and $CD8^+$ cell density correlations are presented separately in the tumor center and the invasive margin. N=913 for CD3 in the tumor center, N=725 for CD3 in the invasive margin, N=877 for CD8 in the tumor center, N=738 for CD8 in the invasive margin.

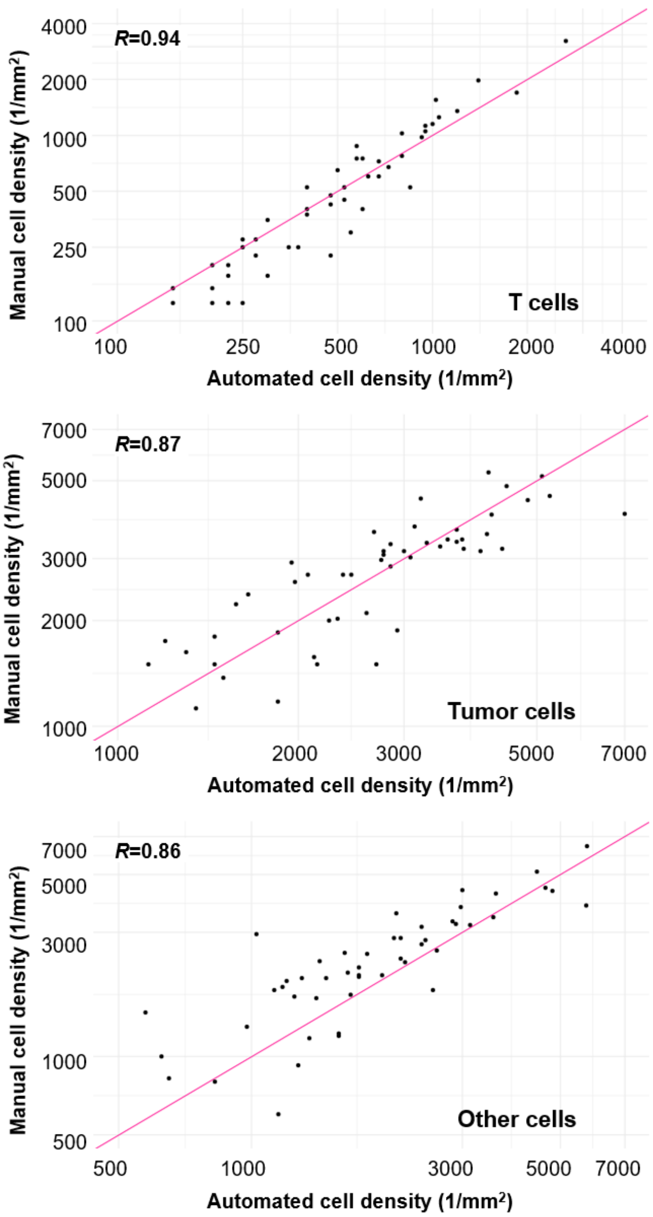

Figure S5 Correlation diagrams of manually and automatedly calculated cell densities measured using Spearman's rank correlation coefficients (*R*). Red line depicts perfect concordance (slope=1). Cell densities were calculated in 50 tumor regions manually and using the optimized, automated method utilizing the QuPath software. The correlations are presented separately for T cells, tumor cells and other cells.

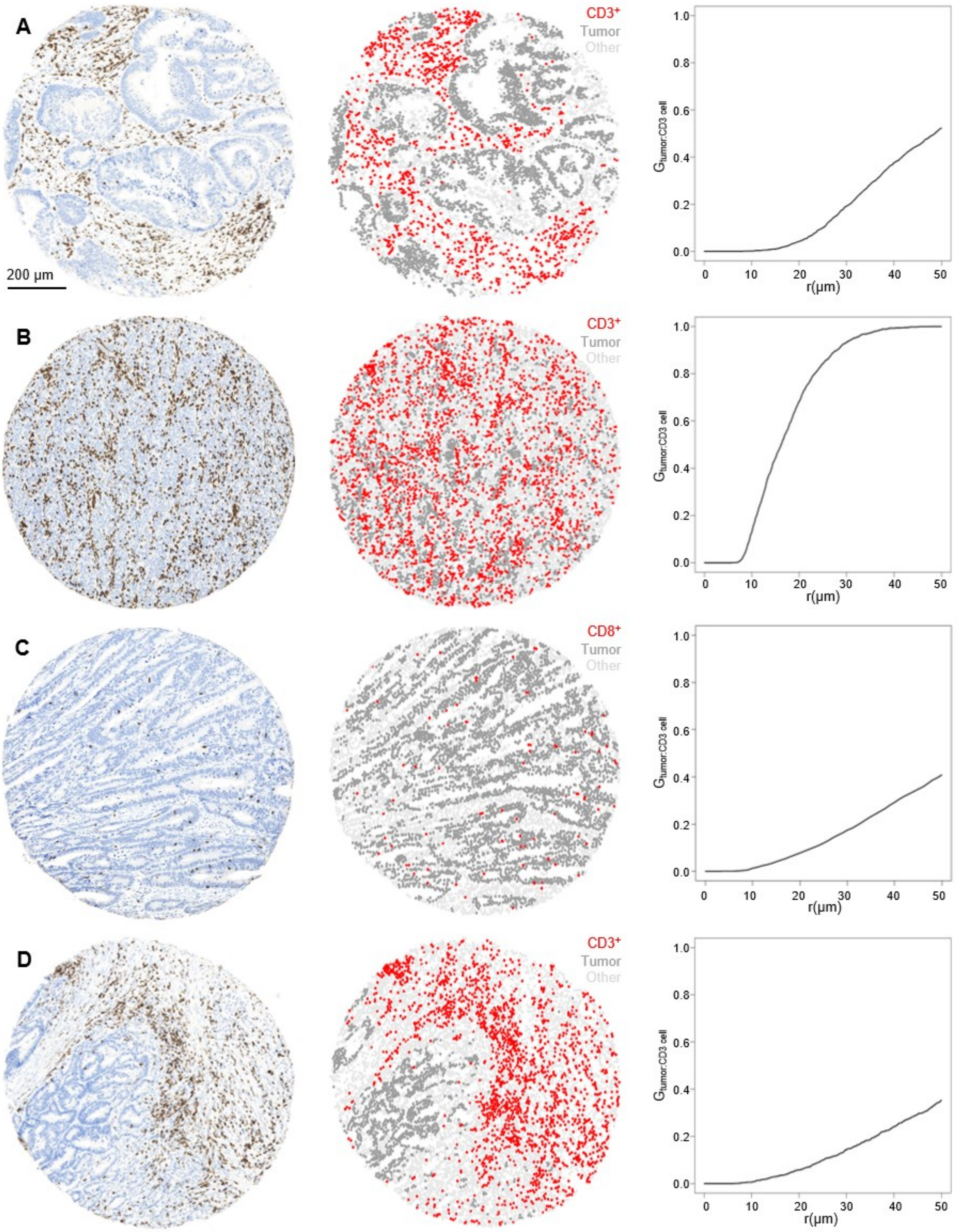

Figure S6 Examples of tissue microarray cores with distinct T cell infiltration patterns. (A-D) $CD3^+$ and $CD8^+$ stained immunohistochemistry images, corresponding phenotyping maps for T cells, tumor cells and other cells, and G-cross ($G_{tumor:T\ cell}$) as a function of radius (r).

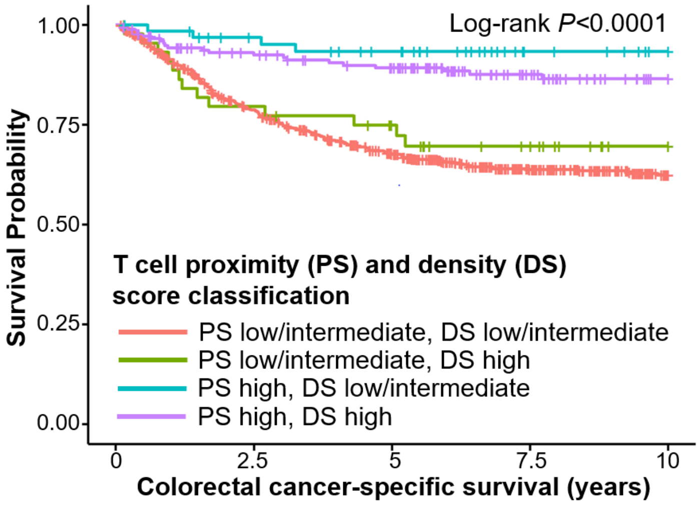

	Years, number at risk				
	0	2.5	5	7.5	10
PS 1-2, DS 1-2	694	500	382	260	160
PS 1-2, DS 3	45	35	28	20	11
PS 3, DS 1-2	65	56	50	32	26
PS 3, DS 3	179	149	135	89	62

Figure S7 Kaplan-Meier cancer-specific survival curves for combined T cell proximity and density score variable. Statistical significance was determined with Log-rank test.

$G_{tumor:T\ cell}$ (20 µm)

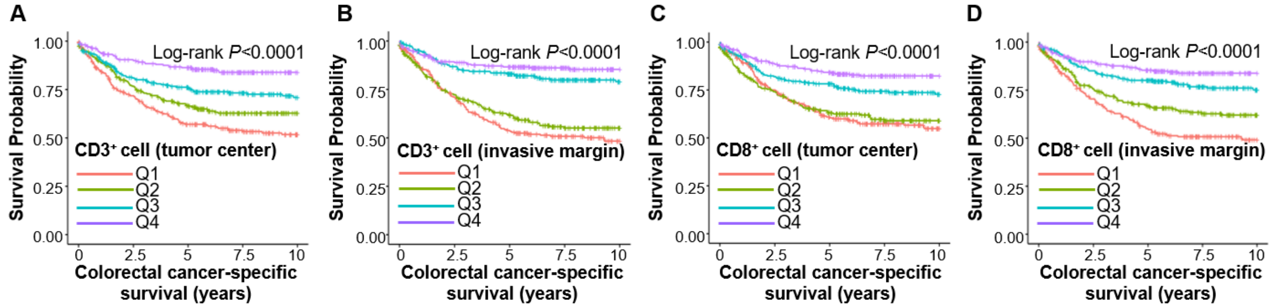

A

	Years, number at risk				
	0	2.5	5	7.5	10
Q1	246	164	116	80	48
Q2	246	182	136	88	49
Q3	246	187	154	109	73
Q4	245	207	189	124	89

B

	Years, number at risk				
	0	2.5	5	7.5	10
Q1	246	168	115	81	48
Q2	246	171	129	83	52
Q3	246	201	173	117	76
Q4	245	200	178	120	83

C

	Years, number at risk				
	0	2.5	5	7.5	10
Q1	246	171	123	79	46
Q2	246	177	130	86	51
Q3	246	191	162	115	74
Q4	245	201	180	121	88

D

	Years, number at risk				
	0	2.5	5	7.5	10
Q1	246	171	123	78	47
Q2	246	170	132	92	60
Q3	246	204	170	112	72
Q4	245	203	177	119	80

T cell density

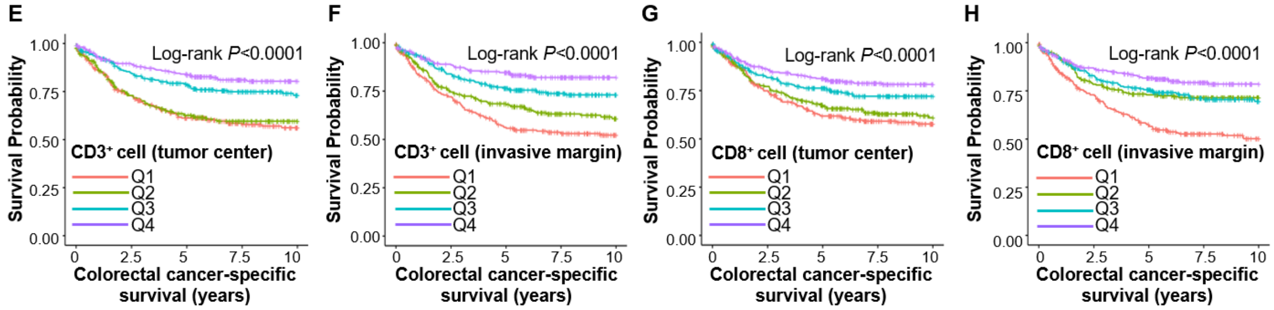

E

	Years, number at risk				
	0	2.5	5	7.5	10
Q1	246	167	121	80	48
Q2	246	171	133	93	58
Q3	246	201	164	111	69
Q4	245	201	177	117	84

F

	Years, number at risk				
	0	2.5	5	7.5	10
Q1	246	172	121	84	51
Q2	246	172	141	91	63
Q3	246	192	152	104	66
Q4	245	204	181	122	79

G

	Years, number at risk				
	0	2.5	5	7.5	10
Q1	246	173	128	84	50
Q2	246	184	135	90	53
Q3	246	187	157	112	70
Q4	245	196	175	115	86

H

	Years, number at risk				
	0	2.5	5	7.5	10
Q1	246	166	119	78	47
Q2	246	171	132	94	62
Q3	246	202	168	111	71
Q4	245	201	176	118	79

Figure S8 Kaplan-Meier cancer-specific survival curves for G-cross ($G_{tumor:T\ cell}$) proximity function values at 20 µm radius and for T cell densities. Analyses were done separately for $CD3^+$ and $CD8^+$ cells in the tumor center and invasive margin by using ordinal quartiles Q1–Q4 (from low to high). Statistical significance was determined with Log-rank test.

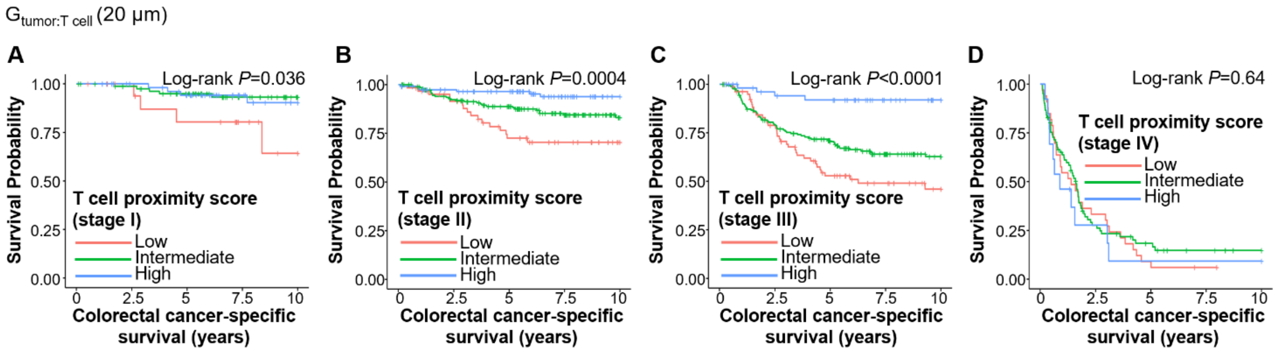

A

	Years, number at risk				
	0	2.5	5	7.5	10
Low	19	16	12	7	3
Intermediate	84	78	68	45	29
High	59	52	45	28	20

B

	Years, number at risk				
	0	2.5	5	7.5	10
Low	62	51	37	27	16
Intermediate	193	165	139	101	59
High	116	104	95	62	46

C

	Years, number at risk				
	0	2.5	5	7.5	10
Low	82	57	34	23	14
Intermediate	184	138	107	72	49
High	56	46	44	30	21

D

	Years, number at risk				
	0	2.5	5	7.5	10
Low	33	11	3	1	0
Intermediate	82	19	10	4	1
High	13	3	1	1	1

Figure S9 Kaplan-Meier cancer-specific survival curves for G-cross ($G_{tumor:T\ cell}$) proximity function values at 20 µm radius. Analyses were done separately for tumors of different disease stage groups I–IV. Statistical significance was determined with Log-rank test.

Supplementary tables

Table S1 Immunohistochemical methods for the detection of T cells and mismatch repair enzyme expression.

Cell type	Antigen	Antigen retrieval	Antibody type	Manufacturer	Clone	Code	Dilution
T cells	CD3	Tris-EDTA pH9⁺	Mouse monoclonal	Leica Biosystems, Buffalo Grove, IL, USA	LN10	PA055	RTU
Cytotoxic T cells	CD8	Tris-EDTA pH9⁺	Rabbit monoclonal	Thermo Fisher Scientific, Waltham, MA, USA	SP16	RM-9116	1:100
MLH1 cells	MLH1	Tris-EDTA pH9⁺	Mouse monoclonal	Novocastra, Leica Biosystems, Nussloch, Germany	ES05	NCL-L-MLH1	1:50
MSH2 cells	MSH2	Tris-EDTA pH9⁺	Mouse monoclonal	Calbiochem, San Diego, CA, United States	FE11	NA27	1:50
MSH6 cells	MSH6	Tris-EDTA pH9⁺	Mouse monoclonal	Epitomics, Burlingame, CA, USA	EP49	AC-0047 EU	1:150
PMS2 cells	PMS2	Tris-EDTA pH9⁺	Mouse monoclonal	BD Biosciences, Pharmingen, San Diego, CA, USA	A16-4	5564151	1:100

Table S2 Immunohistochemical methods utilized in the validation cohort.

Cell type	Antigen	Antigen retrieval	Antibody type	Manufacturer	Clone	Code	Dilution	Incubation	Antibody visualization
T cells	CD3	Tris-EDTA pH9*	Mouse monoclonal	Novocastra, Leica Biosystems, Nussloch, Germany	PS1	NCL-CD3-PS1	1:50	30 min	EnVision
Cytotoxic T cells	CD8	Tris-EDTA pH9*	Mouse monoclonal	Novocastra, Leica Biosystems, Nussloch, Germany	4B11	NCL-CD8-4B11	1:200	30 min	EnVision
MLH1* cells	MLH1	Tris-EDTA pH9*	Mouse monoclonal	Novocastra, Leica Biosystems, Nussloch, Germany	ES05	NCL-L-MLH1	1:100	90 min	EnVision
MSH2* cells	MSH2	Tris-EDTA pH9*	Mouse monoclonal	BD Biosciences, Pharmingen, San Diego, CA, USA	G219-1129	556349	1:200	60 min	EnVision
MSH6* cells	MSH6	Tris-EDTA pH9*	Mouse monoclonal	BD Biosciences, Pharmingen, San Diego, CA, USA	44/MSH6	610919	1:150	90 min	Envision
PMS2* cells	PMS2	Tris-EDTA pH9*	Mouse monoclonal	BD Biosciences, Pharmingen, San Diego, CA, USA	A16-4	5564151	1:100	90 min	EnVision
BRAF V600E mutated cells	BRAF V600E	Ventana CC1	Mouse monoclonal	Spring Bioscience, Pleasonton, CA, US	VE1	E19292	1:2000	32 min	Optiview

*In a microwave oven at 800 W for 2 min and at 150 W for 15 min.

Table S3 Demographic and clinical characteristics of colorectal cancer cases according to T cell density score.

Characteristic	Total N	T cell density score			*P*
		Low	Intermediate	High	
All cases	983 (100%)	163 (17%)	596 (61%)	224 (23%)	
Sex					0.26
Female	479 (49%)	80 (49%)	281 (47%)	120 (54%)	
Male	504 (51%)	83 (51%)	315 (53%)	104 (46%)	
Age (years)					0.33
<65	265 (27%)	46 (28%)	165 (28%)	54 (24%)	
65-75	348 (35%)	55 (34%)	220 (37%)	73 (33%)	
>75	370 (38%)	62 (38%)	211 (35%)	97 (43%)	
Year of operation					0.21
2000-2005	299 (30%)	49 (30%)	174 (29%)	76 (34%)	
2006-2010	315 (32%)	60 (37%)	196 (33%)	59 (26%)	
2011-2015	369 (38%)	54 (33%)	226 (38%)	89 (40%)	
Tumor location					0.003
Proximal colon	478 (49%)	66 (41%)	281 (47%)	131 (59%)	
Distal colon	359 (37%)	67 (41%)	232 (39%)	60 (27%)	
Rectum	146 (15%)	30 (18%)	83 (14%)	33 (15%)	
AJCC disease stage					0.0002
I	162 (16%)	19 (12%)	90 (15%)	53 (24%)	
II	371 (38%)	55 (34%)	220 (37%)	96 (43%)	
III	322 (33%)	64 (39%)	198 (33%)	60 (27%)	
IV	128 (13%)	25 (15%)	88 (15%)	15 (6.7%)	
Tumor grade					0.010
Low-grade (well to moderately differentiated)	813 (83%)	144 (88%)	497 (83%)	172 (77%)	
High-grade (poorly differentiated)	170 (17%)	19 (12%)	99 (17%)	52 (23%)	
Lymphovascular invasion					0.0004
No	772 (79%)	120 (74%)	455 (76%)	197 (88%)	
Yes	211 (21%)	43 (26%)	141 (24%)	27 (12%)	
MMR status					<0.0001
MMR proficient	833 (85%)	150 (92%)	520 (87%)	163 (73%)	
MMR deficient	150 (15%)	13 (8.0%)	76 (13%)	61 (27%)	
BRAF status					0.001
Wild-type	824 (84%)	146 (90%)	504 (85%)	174 (78%)	
Mutant	159 (16%)	17 (10%)	9 (15%)	50 (22%)	

Abbreviations: AJCC, American Joint Committee on Cancer; MMR, mismatch repair.

Table S4 Multivariable Cox regression models of cancer-specific survival and overall survival for T cell proximity and for T cell density scores with all variables.

	T cell proximity score		T cell density score	
	Cancer-specific survival	Overall survival	Cancer-specific survival	Overall survival
	Multivariable HR (95% CI)	Multivariable HR (95% CI)	Multivariable HR (95% CI)	Multivariable HR (95% CI)
T cell proximity score			Not included	Not included
Low	1 (referent)	1 (referent)		
Intermediate	0.72 (0.55-0.94)	0.74 (0.59-0.91)		
High	0.33 (0.20-0.52)	0.57 (0.43-0.76)		
T cell density score	Not included	Not included		
Low			1 (referent)	1 (referent)
Intermediate			0.74 (0.55-0.99)	0.78 (0.67-1.06)
High			0.47 (0.31-0.73)	0.62 (0.46-0.84)
Age				
<65	1 (referent)	1 (referent)	1 (referent)	1 (referent)
65-75	1.12 (0.82-1.53)	1.31 (1.01-1.70)	1.17 (0.86-1.59)	1.32 (1.02-1.70)
>75	1.82 (1.34-2.48)	2.94 (2.31-3.74)	1.83 (1.34-2.49)	2.95 (2.32-3.76)
Gender				
Male	1 (referent)	1 (referent)	1 (referent)	1 (referent)
Female	0.79 (0.61-1.02)	0.69 (0.57-0.83)	0.80 (0.62-1.03)	0.69 (0.57-0.83)
Year of operation				
2000-2005	1 (referent)	1 (referent)	1 (referent)	1 (referent)
2006-2010	0.63 (0.47-0.85)	0.70 (0.56-0.86)	0.63 (0.47-0.84)	0.69 (0.55-0.85)
2011-2015	0.48 (0.35-0.65)	0.59 (0.47-0.74)	0.49 (0.36-0.66)	0.60 (0.47-0.75)
Tumor location				
Proximal colon	1 (referent)	1 (referent)	1 (referent)	1 (referent)
Distal colon	0.80 (0.60-1.06)	0.93 (0.75-1.14)	0.81 (0.61-1.07)	0.93 (0.75-1.14)
Rectum	0.76 (0.52-1.12)	0.88 (0.66-1.17)	0.76 (0.52-1.12)	0.87 (0.65-1.16)
AJCC disease stage				
I-II	1 (referent)	1 (referent)	1 (referent)	1 (referent)
III	2.51 (1.81-3.48)	1.34 (1.08-1.66)	2.61 (1.88-3.62)	1.35 (1.09-1.67)
IV	14.9 (10.5-21.1)	7.40 (5.73-9.56)	16.8 (11.9-23.4)	7.79 (6.04-10.0)
Tumor grade				
Low-grade (well to moderately differentiated)	1 (referent)	1 (referent)	1 (referent)	1 (referent)
High-grade (poorly differentiated)	1.88 (1.37-2.59)	1.89 (1.49-2.40)	1.93 (1.40-2.65)	1.95 (1.53-2.48)
Lymphovascular invasion				
No	1 (referent)	1 (referent)	1 (referent)	1 (referent)
Yes	1.80 (1.38-2.34)	1.53 (1.24-1.89)	1.83 (1.41-2.38)	1.53 (1.24-1.89)
MMR status				
MMR proficient	1 (referent)	1 (referent)	1 (referent)	1 (referent)
MMR deficient	0.58 (0.33-1.00)	0.71 (0.49-1.02)	0.51 (0.30-0.88)	0.67 (0.47-0.97)
BRAF mutation				
Wild-type	1 (referent)	1 (referent)	1 (referent)	1 (referent)
Mutant	1.46 (0.92-2.31)	1.55 (1.11-2.17)	1.40 (0.89-2.21)	1.50 (1.07-2.09)

Abbreviations: AJCC, American Joint Committee on Cancer; CI, confidence interval; HR, hazard ratio; MMR, mismatch repair.

Table S5 Univariable and multivariable Cox regression models for cancer-specific survival and overall survival according to combined T cell proximity score and T cell density score variable.

		Colorectal cancer-specific survival			Overall survival		
	No. of cases	No. of events	Univariable HR (95% CI)	Multivariable HR (95% CI)	No. of events	Univariable HR (95% CI)	Multivariable HR (95% CI)
PS low/intermediate, DS low/intermediate	694	232	1 (referent)	1 (referent)	377	1 (referent)	1 (referent)
PS low/intermediate, DS high	45	13	0.82 (0.47-1.44)	0.88 (0.50-1.57)	19	0.74 (0-46-1.17)	0.81 (0.51-1.29)
PS high, DS low/intermediate	65	4	0.15 (0.06-0.42)	0.28 (0.10-0.77)	23	0.53 (0.35-0.80)	0.71 (0.46-1.10)
PS high, DS high	179	21	0.31 (0.20-0.48)	0.45 (0.28-0.73)	75	0.66 (0.52-0.85)	0.72 (0.55-0.94)
P_{trend}			<0.0001	0.0001		<0.0001	0.0069

The tumors were categorized into the four subgroups: 1) low/intermediate proximity score and low/intermediate density score, 2) low/intermediate proximity score and high density score, 3) high proximity score and low/intermediate density score and 4) high proximity score and high density score.

Abbreviations: CI, confidence interval; DS, T cell density score; HR, hazard ratio; PS, T cell proximity score

Multivariable Cox proportional hazards regression models were adjusted for sex, age (<65, 65–75, >75), year of operation (2000–2005, 2006–2010, 2011–2015), tumor location (proximal colon, distal colon, rectum), tumor grade (well/moderately differentiated, poorly differentiated), lymphovascular invasion (negative, positive), MMR status (proficient, deficient), *BRAF* status (wild-type, mutant).

P_{trend} values were calculated by using the four ordinal categories of T cell proximity and density scores as continuous variables in univariable and multivariable Cox proportional hazard regression models.

Table S6 Univariable and multivariable Cox regression models for cancer-specific and overall survival according to T cell proximity scores at 10–50 µm, 100 µm and 500 µm radii.

		Colorectal cancer-specific survival			Overall survival		
	No. of cases	No. of events	Univariable HR (95% CI)	Multivariable HR (95% CI)	No. of events	Univariable HR (95% CI)	Multivariable HR (95% CI)
T cell proximity score (10 µm)							
Low	182	83	1 (referent)	1 (referent)	123	1 (referent)	1 (referent)
Intermediate	570	164	0.55 (0.42-0.72)	0.77 (0.59-1.02)	279	0.61 (0.49-0.75)	0.74 (0.60-0.93)
High	231	23	0.17 (0.11-0.27)	0.32 (0.20-0.53)	92	0.42 (0.32-0.56)	0.54 (0.40-0.73)
P_{trend}			<0.0001	<0.0001		<0.0001	<0.0001
T cell proximity score (20 µm)							
Low	194	88	1 (referent)	1 (referent)	127	1 (referent)	1 (referent)
Intermediate	545	157	0.57 (0.44-0.75)	0.72 (0.55-0.94)	269	0.66 (0.53-0.81)	0.74 (0.59-0.91)
High	244	25	0.18 (0.12-0.29)	0.33 (0.20-0.52)	98	0.47 (0.36-0.61)	0.57 (0.43-0.76)
P_{trend}			<0.0001	<0.0001		<0.0001	0.0001
T cell proximity score (30 µm)							
Low	194	90	1 (referent)	1 (referent)	130	1 (referent)	1 (referent)
Intermediate	537	155	0.55 (0.42-0.71)	0.70 (0.54-0.92)	264	0.62 (0.50-0.77)	0.69 (0.56-0.86)
High	252	25	0.17 (0.11-0.26)	0.31 (0.19-0.49)	100	0.44 (0.34-0.57)	0.49 (0.37-0.65)
P_{trend}			<0.0001	<0.0001		<0.0001	<0.0001
T cell proximity score (40 µm)							
Low	199	90	1 (referent)	1 (referent)	131	1 (referent)	1 (referent)
Intermediate	536	155	0.56 (0.43-0.72)	0.69 (0.53-0.90)	264	0.63 (0.51-0.78)	0.68 (0.55-0.85)
High	248	25	0.18 (0.11-0.27)	0.32 (0.20-0.52)	99	0.45 (0.35-0.59)	0.55 (0.41-0.73)
P_{trend}			<0.0001	<0.0001		<0.0001	<0.0001
T cell proximity score (50 µm)							
Low	206	92	1 (referent)	1 (referent)	135	1 (referent)	1 (referent)
Intermediate	532	152	0.55 (0.43-0.72)	0.68 (0.52-0.89)	260	0.62 (0.51-0.77)	0.67 (0.54-0.82)
High	245	26	0.19 (0.12-0.29)	0.34 (0.21-0.54)	99	0.46 (0.35-0.60)	0.55 (0.41-0.73)
P_{trend}			<0.0001	<0.0001		<0.0001	<0.0001
T cell proximity score (100 µm)							
Low	186	83	1 (referent)	1 (referent)	120	1 (referent)	1 (referent)
Intermediate	552	156	0.56 (0.43-0.73)	0.68 (0.52-0.89)	279	0.67 (0.54-0.83)	0.71 (0.57-0.88)
High	245	31	0.23 (0.15-0.34)	0.39 (0.25-0.61)	95	0.45 (0.35-0.59)	0.53 (0.40-0.71)
P_{trend}			<0.0001	<0.0001		<0.0001	<0.0001
T cell proximity score (500 µm)							
Low	32	15	1 (referent)	1 (referent)	22	1 (referent)	1 (referent)
Intermediate	436	142	0.66 (0.39-1.12)	1.14 (0.66-1.97)	242	0.74 (0.48-1.14)	1.04 (0.66-1.63)
High	505	113	0.40 (0.23-0.69)	0.92 (0.53-1.62)	230	0.51 (0.35-0.74)	0.91 (0.57-1.43)
P_{trend}			<0.0001	0.22		<0.0001	0.21

Abbreviations: CI, confidence interval; HR, hazard ratio.

Multivariable Cox proportional hazards regression models were adjusted for sex, age (<65, 65–75, >75), year of operation (2000–2005, 2006–2010, 2011–2015), tumor location (proximal colon, distal colon, rectum), disease stage (I–II, III, IV), tumor grade (well/moderately differentiated, poorly differentiated), lymphovascular invasion (negative, positive), MMR status (proficient, deficient), *BRAF* status (wild-type, mutant).

P_{trend} values were calculated by using the three ordinal categories of T cell proximity score and T cell density score as continuous variables in univariable and multivariable Cox proportional hazard regression models.

Table S7 T cell proximity score in strata of MMR status and patient survival.

		Colorectal cancer-specific survival			Overall survival		
	No. of cases	No. of events	Univariable HR (95% CI)	Multivariable HR (95% CI)	No. of events	Univariable HR (95% CI)	Multivariable HR (95% CI)
MMR deficient							
T cell proximity score							
Low	10	4	1 (referent)	1 (referent)	6	1 (referent)	1 (referent)
Intermediate or high	140	23	0.40 (0.14-1.15)	0.51 (0.12-2.12)	72	0.78 (0.34-1.79)	0.93 (0.37-2.36)
P			0.089	0.36		0.56	0.88
MMR proficient							
T cell proximity score							
Low	184	84	1 (referent)	1 (referent)	121	1 (referent)	1 (referent)
Intermediate or high	649	159	0.46 (0.36-0.60)	0.63 (0.48-0.83)	295	0.57 (0.46-0.71)	0.69 (0.56-0.86)
P			<0.0001	0.0012		<0.0001	0.0009
$P_{interaction}$			0.71	0.69		0.48	0.89

Abbreviations: CI, confidence interval; HR, hazard ratio

Multivariable Cox proportional hazards regression models were adjusted for sex, age (<65, 65–75, >75), year of operation (2000–2005, 2006–2010, 2011–2015), tumor location (proximal colon, distal colon, rectum), disease stage (I–II, III, IV), tumor grade (well/moderately differentiated, poorly differentiated), lymphovascular invasion (negative, positive), *BRAF* status (wild-type, mutant).

$P_{interaction}$ was calculated using the Wald test for the cross product of the T cell proximity score (intermediate or high vs low) and MMR status (proficient vs deficient) in the Cox regression model.

Table S8 T cell proximity score in strata of AJCC disease stage and patient survival.

		Colorectal cancer-specific survival			Overall survival		
	No. of cases	No. of events	Univariable HR (95% CI)	Multivariable HR (95% CI)	No. of events	Univariable HR (95% CI)	Multivariable HR (95% CI)
AJCC disease stage (I)							
T cell proximity score							
Low	19	4	1 (referent)	1 (referent)	8	1 (referent)	1 (referent)
Intermediate or high	143	9	0.24 (0.07-0.79)	0.28 (0.08-0.98)	50	0.69 (0.32-1.45)	0.64 (0.29-1.40)
P			0.018	0.046		0.32	0.26
AJCC disease stage (II)							
T cell proximity score							
Low	62	16	1 (referent)	1 (referent)	31	1 (referent)	1 (referent)
Intermediate or high	309	33	0.38 (0.21-0.69)	0.40 (0.22-0.74)	123	0.72(0.49-1.07)	0.69 0.46-1.04)
P			0.002	0.004		0.10	0.076
AJCC disease stage (III)							
T cell proximity score							
Low	80	37	1 (referent)	1 (referent)	55	1 (referent)	1 (referent)
Intermediate or high	242	66	0.50 (0.33-0.75)	0.50 (0.32-0.76)	106	0.53 (0.38-0.73)	0.51 (0.36-0.72)
P			0.0008	0.001		0.0001	0.0002
AJCC disease stage (IV)							
T cell proximity score							
Low	33	74	1 (referent)	1 (referent)	33	1 (referent)	1 (referent)
Intermediate or high	95	31	0.93 (0.61-1.41)	1.10 (0.68-1.80)	88	1.01 (0.68-1.51)	1.34 (0.85-2.13)
P			0.73	0.69		0.95	0.21

Abbreviations: AJCC, American Joint Committee on Cancer; CI, confidence interval; HR, hazard ratio.
Multivariable Cox proportional hazards regression models were adjusted for sex, age (<65, 65–75, >75), year of operation (2000–2005, 2006–2010, 2011–2015), tumor location (proximal colon, distal colon, rectum), tumor grade (well/moderately differentiated, poorly differentiated), lymphovascular invasion (negative, positive), MMR status (proficient, deficient), *BRAF* status (wild-type, mutant).

Table S9 T cell proximity score in strata of AJCC disease stage and patient survival.

		Colorectal cancer-specific survival			Overall survival		
	No. of cases	No. of events	Univariable HR (95% CI)	Multivariable HR (95% CI)	No. of events	Univariable HR (95% CI)	Multivariable HR (95% CI)
Low AJCC disease stage (I-III)							
T cell proximity score							
Low	161	57	1 (referent)	1 (referent)	94	1 (referent)	1 (referent)
Intermediate or high	694	108	0.38 (0.27-0.52)	0.40 (0.29-0.56)	279	0.58 (0.45-0.72)	0.57 (0.45-0.73)
P			<0.0001	<0.0001		<0.0001	<0.0001
High AJCC disease stage (IV)							
T cell proximity score							
Low	33	31	1 (referent)	1 (referent)	33	1 (referent)	1 (referent)
Intermediate or high	95	74	0.93 (0.61-1.41)	1.10 (0.68-1.80)	88	1.01 (0.68-1.51)	1.34 (0.85-2.13)
P			0.73	0.69		0.95	0.21
$P_{interaction}$			0.019	<0.0001		0.019	0.001

Abbreviations: AJCC, American Joint Committee on Cancer; CI, confidence interval; HR, hazard ratio.
Multivariable Cox proportional hazards regression models were adjusted for sex, age (<65, 65–75, >75), year of operation (2000–2005, 2006–2010, 2011–2015), tumor location (proximal colon, distal colon, rectum), tumor grade (well/moderately differentiated, poorly differentiated), lymphovascular invasion (negative, positive), MMR status (proficient, deficient), *BRAF* status (wild-type, mutant).
$P_{interaction}$ was calculated using the Wald test for the cross product of the T cell proximity score (intermediate or high vs low) and disease stage (stage IV vs stage I–III) in the Cox regression model.

Table S10 Demographic and clinicopathologic characteristics of colorectal cancer validation cohort cases according to T cell proximity score.

		T cell proximity score			
Characteristic	Total N	Low	Intermediate	High	*P*
All cases	246 (100%)	32 (17%)	153 (62%)	61 (25%)	
Sex					
Female	124 (50%)	17 (53%)	79 (52%)	28 (46%)	0.71
Male	122 (50%)	15 (47%)	74 (48%)	33 (54%)	
Age (years)					
<65	75 (30%)	12 (38%)	44 (29%)	19 (31%)	0.21
65-75	86 (35%)	12 (38%)	59 (39%)	15 (25%)	
>75	85 (35%)	8 (25%)	50 (33%)	27 (44%)	
Year of operation					
2006-2009	98 (40%)	12 (38%)	69 (45%)	17 (28%)	0.064
2010-2014	148 (60%)	20 (63%)	84 (55%)	44 (72%)	
Tumor location					
Proximal colon	107 (43%)	13 (41%)	64 (42%)	30 (49%)	0.51
Distal colon	64 (26%)	11 (34%)	37 (24%)	16 (26%)	
Rectum	75 (30%)	8 (25%)	52 (34%)	15 (25%)	
AJCC disease stage[A]					
I	51 (21%)	0 (0%)	33 (22%)	18 (30%)	<0.0001
II	84 (34%)	10 (31%)	51 (33%)	23 (38%)	
III	71 (29%)	7 (22%)	49 (32%)	15 (25%)	
IV	39 (16%)	15 (47%)	20 (13%)	4 (6.7%)	
Tumor grade[A]					
Low-grade (well to moderately differentiated)	218 (89%)	30 (97%)	136 (89%)	52 (85%)	0.25
High-grade (poorly differentiated)	27 (11%)	1 (3.2%)	17 (11%)	9 (15%)	
Lymphovascular invasion[B]					
No	125 (51%)	6 (19%)	80 (53%)	39 (65%)	0.0002
Yes	118 (49%)	25 (81%)	72 (47%)	21 (35%)	
MMR status[A]					
MMR proficient	212 (87%)	31 (100%)	139 (91%)	42 (69%)	<0.0001
MMR deficient	33 (13%)	0 (0%)	14 (9.2%)	19 (31%)	
BRAF status					
Wild-type	220 (89%)	31 (97%)	143 (94%)	46 (75%)	0.0002
Mutant	26 (11%)	1 (3.1%)	10 (6.5%)	15 (25%)	

Abbreviations: AJCC, American Joint Committee on Cancer; MMR, mismatch repair. *P* values are based on the comparison of categorical data between the ordinal categories of T cell proximity score by the Chi-square test.

[A] Data missing for one case

[B] Data missing for three cases.

Table S11 Univariable and multivariable Cox regression models for cancer-specific survival and overall survival according to T cell proximity score and T cell density score in the validation cohort.

		Colorectal cancer-specific survival			Overall survival		
	No. of cases	No. of events	Univariable HR (95% CI)	Multivariable HR (95% CI)	No. of events	Univariable HR (95% CI)	Multivariable HR (95% CI)
**T cell proximity score**							
Low	32	18	1 (referent)	1 (referent)	19	1 (referent)	1 (referent)
Intermediate	153	35	0.29 (0.17-0.52)	0.42 (0.22-0.81)	52	0.42 (0.25-0.71)	0.54 (0.31-0.98)
High	61	5	0.10 (0.04-0.27)	0.15 (0.05-0.45)	9	0.17 (0.08-0.38)	0.22 (0.09-0.54)
P_{trend}			<0.0001	<0.0001		<0.0001	0.0006
**T cell density score**							
Low	12	7	1 (referent)	1 (referent)	10	1 (referent)	1 (referent)
Intermediate	150	41	0.29 (0.13-0.64)	0.50 (0.20-1.26)	55	0.26 (0.13-0.51)	0.32 (0.15-0.72)
High	84	10	0.12 (0.04-0.31)	0.23 (0.07-0.73)	15	0.12 (0.05-0.27)	0.18 (0.07-0.45)
P_{trend}			<0.0001	0.0010		<0.0001	0.0008

Abbreviations: CI, confidence interval; HR, hazard ratio

Multivariable Cox proportional hazards regression models were adjusted for sex, age (<65, 65–75, >75), year of operation (2006–2009, 2010–2014), tumor location (proximal colon, distal colon, rectum), disease stage (I–II, III, IV), tumor grade (well/moderately differentiated, poorly differentiated), lymphovascular invasion (negative, positive), mismatch repair (MMR) status (proficient, deficient), *BRAF* status (wild-type, mutant).

P_{trend} values were calculated by using the three ordinal categories of T cell proximity score and T cell density score as continuous variables in univariable and multivariable Cox proportional hazard regression models.

Table S12. Comparison of prognostic power of T cell proximity score and T cell density score using Cox regression models for cancer-specific survival in the validation cohort.

	No. of cases	No. of events	Model 1 (univariable) HR (95% CI)	Model 2 (multivariable) HR (95% CI)	Model 3 (multivariable) HR (95% CI)
**T cell proximity score**					
Low	32	18	1 (referent)	1 (referent)	1 (referent)
Intermediate	153	35	0.29 (0.17-0.52)	0.36 (0.19-0.67)	0.42 (0.19-0.90)
High	61	5	0.10 (0.04-0.27)	0.15 (0.05-0.48)	0.16 (0.04-0.66)
P_{trend}			<0.0001	0.0002	0.0068
**T cell density score**					
Low	12	7	1 (referent)	1 (referent)	1 (referent)
Intermediate	150	41	0.29 (0.13-0.64)	0.45 (0.19-1.04)	1.09 (0.36-3.32)
High	84	10	0.12 (0.04-0.31)	0.33 (0.11-1.02)	0.97 (0.21-4.41)
P_{trend}			<0.0001	0.088	0.90

Abbreviations: CI, confidence interval; HR, hazard ratio.
Model 2: Cox proportional hazards regression model including T cell proximity score and T cell density score.
Model 3: Cox proportional hazards regression model based on Model 2 that was additionally adjusted for sex, age (<65, 65–75, >75), year of operation (2006–2009, 2010–2014), tumor location (proximal colon, distal colon, rectum), disease stage (I–II, III, IV), tumor grade (well/moderately differentiated, poorly differentiated), lymphovascular invasion (negative, positive), mismatch repair (MMR) status (proficient, deficient), *BRAF* status (wild-type, mutant).
P_{trend} values were calculated by using the three ordinal categories of T cell proximity score and T cell density score as continuous variables in univariable and multivariable Cox proportional hazard regression models.
